# Gm614 Protects Germinal Center B Cells From Death by Suppressing Caspase-1 Transcription in Lupus-Prone Mice

**DOI:** 10.3389/fimmu.2020.585726

**Published:** 2020-10-21

**Authors:** Youdi He, Ruonan Xu, Bing Zhai, Shan Zhou, Xiaoqian Wang, Renxi Wang

**Affiliations:** ^1^Beijing Institute of Brain Disorders, Laboratory of Brain Disorders, Ministry of Science and Technology, Collaborative Innovation Center for Brain Disorders, Capital Medical University, Beijing, China; ^2^Department of Neurology, Beijing Chaoyang Hospital, Capital Medical University, Beijing, China; ^3^Department of Geriatric Hematology, Nanlou Division, Chinese People’s Liberation Army of China General Hospital, National Clinical Research Center for Geriatric Diseases, Beijing, China; ^4^Staidson (Beijing) Biopharmaceuticals Co., Ltd, Beijing, China

**Keywords:** Gm614, lupus, GC B cells, cell death, caspase-1

## Abstract

Only a few signaling pathways have been reported in germinal center (GC) B-cell proliferation and death. In this study, we showed that a novel uncharacterized Gm614 protein is highly expressed in GC B cells from lupus-prone mice. Critically, ablation of this GC B-cell-specific Gm614 promoted GC B-cell death and mitigation of autoimmune symptoms, whereas overexpression protected GC B cells from death and exacerbated autoimmune symptoms. We demonstrated that mechanistically, nuclear-localized Gm614 reduced caspase-1 expression in GC B cells by binding with caspase-1 promoter to suppress its activation. Our results suggest that Gm614 protects GC B cells from death by suppressing caspase-1 transcription in autoimmune diseases. This may provide some hints for targeting the cell proliferation involved in autoimmune diseases.

## Introduction

B cells play an important role in autoimmune diseases. They can present antigen (Ag) to T cells, secrete inflammatory cytokines, and differentiate into autoantibody-producing plasma cells (PCs) (1). B cells are regarded as a valid target for autoimmune diseases, and depletion of B cells with rituximab has proven to be an effective treatment of autoimmune diseases (2). Further exploration of potential B-cell targets may open up new therapeutic opportunities.

Most autoimmune diseases involve an imbalance of B-cell proliferation and death. Specifically, lymphoproliferative syndrome illustrates the role of cell death in autoimmune diseases with natural mutants such as a homozygous null FAS ligand (FASLG) leading to uncontrolled proliferation of lymphocytes accompanied by autoimmune cytopenia (3). Lupus-prone MRL/lpr mice demonstrate similar autoimmune symptoms as systemic lupus erythematosus (SLE) due to the lymphoproliferation-producing spontaneous mutation of Fas (4). Although the parental strains display limited autoimmunity, NZB/W F1 mice develop inflammation-driven lupus-like features similar to that of human lupus patients (5). Therefore, exploring the mechanisms underlying disregulation involved in B-cell death and proliferation will be important in finding effective B-cell-targeting agents for autoimmune diseases.

Germinal centers (GC) may be important sites of immune dis-regulation in autoimmune diseases (6). Spontaneous GC formation in the spleens of several autoimmune mouse strains promote the development of a lupus-like disease (6), and ectopic GC-like structures are also involved in autoimmune diseases such as SLE (7). B cells with a GC phenotype (B220^+^PNA^+^) have been found in the kidneys of an SLE mouse model (8). In GC, Ag-activated B cells undergo a proliferation phase in the GC dark zone (DZ), followed by a non-proliferative selection stage in the GC light zone (LZ) where B cells are selected to differentiate into memory B cells or PCs or die (9).

The signaling pathways involved in germinal center (GC) B-cell proliferation and death are largely unknown. In the present study, Gm614 was shown to be highly expressed in GC B cells from lupus-prone mice. Importantly, our results suggest that Gm614 protects GC B cells from death by suppressing caspase-1 transcription in autoimmune diseases.

## Materials and Methods

### Ethics Committee Approval

Care, use, and treatment of mice in this study were in strict agreement with international guidelines for the care and use of laboratory animals. This study was approved by the Animal Ethics Committee of Beijing Institute of Brain Disorders.

### Mice and Immunization

All mice used in the study are on the C57BL/6 background. Seven-week-old female C57BL/6 mice were purchased from the Chinese Academy of Medical Sciences (Beijing, China). Lupus-prone MRL/MpJ/lpr/lpr (MRL/lpr) mice and age-matched MRL/MpJ/+/+ (MRL/+) mice (Nanjing Biomedical Research Institute of Nanjing University, Nanjing, China) have been previously described (10). Other lupus-prone NZB/WF1 mice (New Zealand Black/New Zealand White F1 mice) and their control NZW/LacJ mice were purchased from Chinese Academy of Medical Sciences, Beijing, China, and have been previously described (11). Gm614^fl/fl^ (Gm614^F/F^) mice were developed by Cyagen Biosciences Inc., Guangzhou, China. C*γ*1-cre (C*γ*1^cre^) (B6.129P2[Cg]-Ighg1tm1[IRES-cre]Cgn/J) mice as a tool to mediate GC-specific knocked-out of a gene (12, 13) were purchased from Shanghai Biomodel Organism Science & Technology Development Co., Ltd. (Shanghai, China). Gm614^F/F^ mice and C*γ*1^cre^ mice were crossed to obtain GC-specific Gm614-null (C*γ*1^cre^Gm614^F/F^, Gm614 cKO) animals. Gm614 transgenic (Tg) mice were developed by Cyagen Biosciences Inc., Guangzhou, China. BM Chimeras have been used to develop B-specific depletion/expression of a gene (14, 15). μMT mice (B6.129S2-Ighmtm1Cgn/J, Stock No: 002288, The Jackson Lab) were irradiated (1150 cGy) and reconstituted with BM from μMT mice (80%) and Gm614 Tg mice (20%). Thus, 80% of the hematopoietic cells (except B cells) in the chimeric mice will be wild-type in gene expression whereas the B cells can only be derived from Gm614 Tg precursors. The reconstituted mice were named as B^Gm 614 Tg^ mice. B^non Tg^ mice were used as the control. To induce lupus, about 9-week-old mice were injected intraperitoneally (i.p.) with 0.5 ml pristane (2,6,10,14-Tetramethylpentadecane, Cat no. 138462500, Fisher Scientific Corp., USA). In some experiments, mice were injected i.p. with 100 μg of the protein antigen KLH (Keyhole limpet haemocyanin; Sigma, St Louis, MO) in a volume of 0.2 mL of saline.

### Cell Staining, Flow Cytometric Analysis, and Cell Sorting

All cell experiments were strictly prepared on ice, unless otherwise stated in other specific procedures. Cells (1×10^6^ cells/sample) were washed with fluorescence-activated cell sorting staining buffer (phosphate-buffered saline, 2% fetal bovine serum or 1% bovine serum albumin, 0.1% sodium azide). All samples were incubated with anti-Fc receptor Ab (clone 2.4G2, BD Biosciences, San Jose, CA), prior to incubation with other Abs diluted in fluorescence activated cell sorting buffer supplemented with 2% anti-Fc receptor Ab. The samples were filtered immediately before analysis or cell sorting to remove any clumps. The following antibodies were used: PerCP-conjugated anti-mouse B220 (Invitrogen, clone no. RA3-6B2), PE or FITC-conjugated anti-mouse CD19 (Invitrogen, clone no. MB19-1), APC-conjugated anti-mouse IgM (Invitrogen, clone no. 11/41), FITC-conjugated anti-mouse IgD (Invitrogen, clone no. 11-26c), APC or PE-conjugated anti-mouse CD38 (Invitrogen, clone no. 90), FITC-conjugated anti-mouse GL7 (Invitrogen, clone no. GL-7), APC-conjugated anti-mouse CD138 (Invitrogen, clone no. DL-101), PE-conjugated anti-mouse TACI (Invitrogen, clone no. ebio8F10-3), eFluor 450 or PE-conjugated anti-mouse CXCR4 (Invitrogen, clone no. 2B11), and APC-conjugated anti-mouse CD86 (Invitrogen, clone no. GL1). Based on previous studies (16–18), immature B cells (CD19^+^B220^+^IgM^+^IgD^-^), mature B cells (CD19^+^B220^+^IgM^+^IgD^+^), GC B cells (CD19^+^B220^+^GL7^+^CD38^low^), PBs (TACI^+^CD138^+^B220^int^CD19^int^), and PCs (TACI^+^CD138^+^B220^-^CD19^-^) were sorted by flow cytometry (FACS). Data collection and analyses were performed on a FACS Calibur flow cytometer using CellQuest software.

### B Cell Isolation and RNA Sequencing

Splenic B220^+^ B cells were separated by B220 microbeads (Cat No. 130-049-501, Miltenyi Biotec). The transcripts in cells were determined by RNA-sequencing using previous methods (19–22). Briefly, RNeasy Mini Kit (Qiagen, Venlo, Netherlands) was used to isolate and purify total RNA from cells. NanoDrop^®^ND-1000 spectrophotometer and Agilent 2100 Bioanalyzer and RNA 6000 NanoChips (Agilent, Palo Alto, CA, USA) were used to determine RNA concentration and quality, respectively. TruSeq Stranded Total RNA Library Prep Kit with Ribo-Zero Gold (Illumina) was used to prepare Libraries. Transcripts were analyzed by RNA-sequencing (Genewiz Corp., Suzhou, China).

### iTraq Quantitative Proteomics

Each sample of 200 μg total protein in 200 μL denaturing buffer was reduced with 2 μL 1 M DTT at 60 °C for 1 h, and then the cysteine residues were blocked by adding 10 μL 1 M IAA for 40 min at room temperate under dark conditions. The reduced and alkylated protein mixtures were subjected to the FASP protocol with spin ultra-filtration units containing a nominal molecular weight cutoff of 10,000 Da (Sartorius, Gottingen, Germany) and centrifuged at 12,000 rpm for 20 min. The bottom solution was then discarded. After washing with 100 μL dissolution buffer three times, the protein digestions were conducted by incubating the proteins and the Sequencing Grade Modified Trypsin (Promega Corporation, WI, USA) in a 1:50 ratio (trypsin-to-protein mass) at 37 °C overnight. After digestion, the liberated peptides were collected by centrifugation at 12,000 rpm for 20 min and the filtration units were washed with 50 μL of UA buffer. The resultant peptide mixture samples were labeled using an 8-Plex iTRAQ Reagent Kit from Applied Biosystems (Thermo Fisher Scientific Inc., DE, USA) as follows: E2_1: 113; E2_2: 114; Glucose_1: 117; Glucose_2: 118. The labeled peptide mixtures (E2_1: 113 and E2_2: 114, Glucose_1: 117 and Glucose_2: 118) were then pooled together and dried by SpeedVac.

### Quantitative PCR Analysis

Total RNA was extracted from cells with Trizol (Invitrogen Life Technologies). The final RNA pellets were dissolved in 0.1 mM EDTA (2 μl/mg original wet weight). Reverse transcription reactions were carried out on 22 μl of sample using superscript II RNA H-Reverse Transcriptase (Invitrogen Life Technologies) in a reaction volume of 40 μl. All samples were diluted in 160 μl nuclease-free water. qPCR was employed to quantify mouse gene expression from the cDNA samples. Mouse gene expression was normalized to the levels of the β-actin gene.

### Production of Anti-Gm614 Antibody

The production of the polyclonal antibody in Rabbit has been previously described (19, 23). Briefly, The C-terminal end of mouse Gm614 (sequence: SPKGGGAQQTTST SSQLKSKGGR) corresponding to amino acids 144–166 was chosen for peptide synthesis. The peptide was coupled to keyhole limpet and used for the immunization of rabbits and production of anti-Gm614. The polyclonal antibody was purified by protein A chromatograph, and its reactivity with recombinant mouse Gm614 was confirmed by ELISA.

### Western Blot Analysis

On a 10% SDS–polyacrylamide gel, 25 µg of cell protein from whole-cell lysates was electrophoretically separated and then transferred to a PVDF membrane. This membrane was blocked for 1 hour in the solution with 5% fat-free dry milk in Tris-buffered saline containing 0.1% Tween-20 (TBS-T) at room temperature. The blots were then incubated overnight at 4°C with rabbit antibodies against caspase-1 (#24232, Cell Signaling Tech), Gm614 (described above), and β-actin (#4970, Cell Signaling Tech) antibodies diluted 1:1000 in TBS-T containing 5% bovine serum albumin. The membrane was washed for 5 min every time and totally for 4 times with TBS-T, and incubated for 45 min at room temperature with HRP (horseradish peroxidase)-conjugated secondary antibody F(ab’)2 (Zymed Laboratories, San Francisco, CA) (1:20 000 in TBS-T containing 5% bovine serum albumin). Finally, the ECL detection system (Amersham, Arlington Heights, IL) was used to show the protein band.

### Hematoxylin and Eosin (H&E) Staining and Histopathology Scoring

H&E staining has been described previously (24, 25). Kidney specimens were fixed in formalin for 24 h, dehydrated by successive incubation in 70%, 80%, and 90% ethanol for 3 h, and then 100% ethanol I for 2 h, 100% ethanol II for 2 h, and vitrified with xylene I and xylene II for 20 min. After immersing in paraffin I and II for 40 min, the specimens were embedded and sliced. Staining was performed as follows: hematoxylin staining for 15 min, followed by hydrochloric acid alcohol solution for 35 s, eosin staining for 10 min, followed by 90% ethanol for 40 s. Neutral balsam was then used for mounting and the section was observed and photographed by light microscope.

Histopathology was scored based on a standard method described previously (26). Briefly, loss of brush border, grading of tubular necrosis, tubular dilatation, and cast formation in 10 randomly chosen, non-overlapping fields (200 X) were used to score: 0 (none), 1 (≤10%), 2 (11∼25%), 3 (26∼45%), 4 (46∼75%), and 5 (≥76%).

### ELISA Assay

Blood was taken from vena cava with syringe and carefully put in a 1.5 mL E-tube. The tube was placed for 30 min at room temperature (RT) for clotting and then centrifuged for 10 min at 10000 rpm in RT. Sera in the supernatant were collected and tested by ELISA against the following recombinant proteins: Ro60, La, and Sm/RNP (Euroimmun AG, Lübeck, Germany, and Orgentec Diagnostics GmbH, Mainz, Germany), dsDNA (Sigma) in the presence of histone (Sigma), or KLH (Sigma). Goat anti-rat polyvalent HRP-labeled anti-IgG and -IgM antibodies were used as the secondary antibodies (Sigma, SAB3700666-2MG).

### Propidium Iodide (PI)/FACS Analysis

Cell cycle and death analysis has been described previously (19, 23). Briefly, cells were collected and washed 1 time with 5–10 ml of 1×PBS. Cells were suspended in 500 μl 1× PBS containing + 0.1% glucose (at 4°C), and 5 ml of cold 70% EtOH (kept at −20°C) was immediately added, mixed, and kept at 4°C for 1 h. Cell were then spun down and washed once with 1× PBS (10 ml). Without adding more PBS, cells were then spun again for 2 min so that the residual PBS could be removed and cells were then suspended in 300 μl 69 μM PI (propidium iodide, Cat# 537059, Calbiochem, San Diego, CA) solution with 38 mM Na Citrate (Cat# C7254 Sigma, St. Louis, MO) and 20 μl of 10 mg/ml RNase (Cat# R4875, Calbiochem, San Diego, CA). Cells were mixed, incubated at 37°C for 30–45 min, and analyzed by FACS.

### Immunofluorescence and Confocal Microscopy

Cells were seeded onto glass coverslips in 24-well plates, washed with PBS, fixed in 4% formaldehyde solution for 30 min, and permeabilized for 5 min with 0.5% Triton X-100 in TBS. After extensive wash, cells were blocked with 2% BSA in PBS for 30 min. Coverslips were incubated with DAPI and observed under a confocal laser scanning microscope (Leica TCS-SP2). Images were directly captured, saved, and transferred to Adobe Photoshop 5.5.

### Lentiviruses Were Infected Into GC B Cells

cDNA encoding Gm614 full length (1–191), NLS (172–191), full length with AA (176–177) mutation, or (d) full length with AA (188–189) mutation was cloned into EGFP-expressing pReceiver-Lv122 (Fugene Corp., Guangzhou, China) to express an EGFP fusion protein. EGFP fusion protein-expressing LV122 and HIV-based lentiviral packaging plasmids (LT003, Fugene Corp) were co-transfected into 293Ta lentiviral packaging cell line (LT008, Fugene Corp). Cells were cultured for 3 days, and supernatant was collected. Quick and simple concentration of lentiviral particles was done by using a kit (LT007, Fugene Corp). qRT-PCR-based lentiviral titration (LT006, Fugene Corp) was used to determine the copy numbers of HIV lentiviral particles. EGFP fusion protein-expressing LV122-contained lentiviruses were added into CD19^+^B220^+^CD38^lo^GL7^hi^ GC B cell culture.

### Chip-Sequencing Assay

Chromatin immunoprecipitation (Chip) analysis has been described previously (27). Briefly, cells were cross-linked with 1% formaldehyde for 10 min at RT, quenched by the addition of glycine to a final concentration of 0.125 M, and frozen. Chip was performed on two independent pools of cells, each comprising 3 samples. Cell lysis and nuclei isolation was performed using the TruChIP High Cell Chromatin Shearing Kit with SDS (Covaris). Nuclei were sonicated in the S220 Ultrasonicator (Covaris) in order to obtain chromatin fragments ranging from 200 to 500 bp. Sheared chromatin from about 5-10 ×10^6^ cells was incubated overnight with 4 µg of anti-EGFP (Abcam). Protein A magnetic beads were then added for 4 hr incubation followed by sequential washes at increasing stringency and reverse cross-linking. Upon RNAse and proteinase K treatment, Chip DNA was purified using the MiniElute Reaction Cleanup Kit (Qiagen) and quantified using the Quant-iT PicoGreen dsDNA Reagent (Life Technologies). Chip-seq library preparation, sequencing, and analysis were done by IGenecode.

### Caspase-1 Promoter Reporting Gene Analysis

Promoter reporting gene analysis has been described previously (20, 22). Briefly, 0.5 µg firefly luciferase reporter plasmid pEZX-PG04.1 (Fugene Corp., Guangzhou, China) with caspase-1 promoter (-2000 ∼ +100 bp of mouse caspase-1 gene) (Full length), caspase-1 promoter with the deletion of -1612 ∼ -1601 (Δ -1612 ∼ -1601) or -1273 ∼ -1262 (Δ -1273 ∼ -1262), 0.5 µg Gm614-expressing LV201 (Gm614) or empty LV 201 (Vector) plasmid, and 0.05 µg Renilla luciferase reporter vector pRL-SV-40 vector (cat# E2231, Promega Corp.) were co-transduced into 4 X 10^5^ 293T cells in 12-well plate by using 6 µL Lipofectamine^®^2000 Reagent (Cat# 11668-019, Invitrogen Corp.). On day 3, sequential measurement of firefly luciferase (Reporter #1) followed by Renilla luciferase activity (Reporter #2) was assessed on 1420 Multilabel Counter (1420 Victor 3, PerkinElmer Corp.) and analyzed. The results were shown as the ratio of firefly to Renilla luciferase activity.

### Statistics

Statistics were analyzed by using GraphPad Prism (version 5.0, GraphPad Software Inc., USA). The data were shown as mean ± standard error of the mean (SEM). Student’s t test was employed to determine significance between two groups (paired or unpaired) and One-Way or Two-Way ANOVA analysis was used to determine significance among several groups. Differences were considered statistically significant when p < 0.05.

## Results

### Gm614 Expression Was Increased in Germinal Center (GC) B Cells of Lupus-Prone Mice

Our previous studies have shown that Atacicept (TACI-IgG) achieves similar clinical results to belimumab by reducing the levels of circulating mature and activated B cells in the lupus-prone mice (11, 28). To explore the molecular mechanisms, the transcripts in B220^+^ B cells from TACI-IgG-treated lupus-prone mice were assessed using Affymetrix Microarrays described in our previous study (20). We determined gene Gm614 resulted in 0.16, 0.12, and 0.14 fold down-regulation in three independent experiments shown in **Supplementary Tables 1, 2**, and **3**, respectively. These results suggest that Gm614 may be associated with lupus progression.

**Figure 1:**
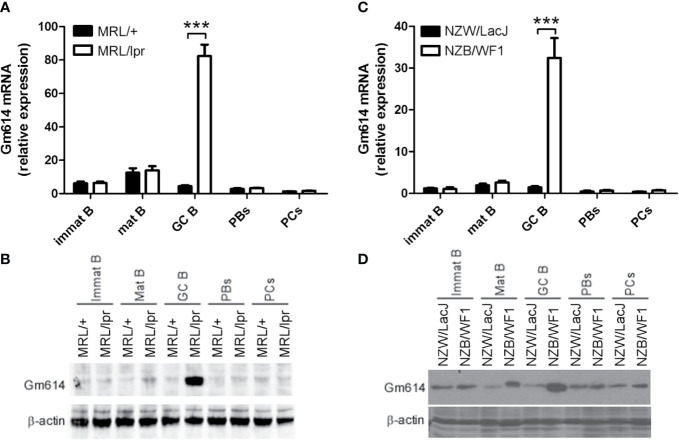
Gm614 was highly expressed in germinal center (GC) B cells from lupus-prone mice. Immature B cells (Immat B), mature B cells (Mat B), GC B cells, plasmablasts (PBs), and plasma cells (PCs) from splenocytes of six lupus-prone MRL/lpr mice and their control MRL/+ mice **(A, B)** or lupus-prone NZB/WF1 mice and their control NZW/LacJ mice **(C, D)** were sorted by flow cytometry (FACS), and subjected to qPCR **(A, C)** and western blot **(B, D)** analysis. **(A–D)** Data represent three independent experiments. **(A, C)** Two-Way ANOVA were followed by Bonferroni post-tests (n = 6), Error bars, s.e.m., ***p < 0.001.

To determine Gm614 expression in B-cell subpopulations in lupus-prone mice, flow cytometry (FACS) was used to sort immature B cells, mature B cells, GC B cells, PBs, and PCs from splenocytes of lupus-prone MRL/lpr **(****Figures 1A, B****)** and NZB/WF1 **(****Figures 1C, D****)** mice. qPCR **(****Figures 1A, C****)** and western blot **(****Figures 1B, D****)** analysis showed that both MRL/lpr and NZB/WF1 mice have higher Gm614 expression in GC B cells compared to the control. These results indicate that lupus-up-regulated Gm614 expression in GC B cells is a more universal inflammation-driven phenomenon.

### Gm614 Knocked-Out (KO) in GC B Cells Mitigated Autoimmune Symptoms

To explore the effect of Gm614 KO on lupus symptoms, we developed C*γ*1^cre^Gm614^fl/fl^ (Gm614 cKO) mice **(****Supplementary Figure 1****)**. Our data showed that Gm614 expression was knocked-out in GC B cells from Gm614 cKO mice **(****Supplementary Figure 1****)**. However, Gm614 cKO mice did not demonstrated any open autoimmune symptoms.

**Figure 2:**
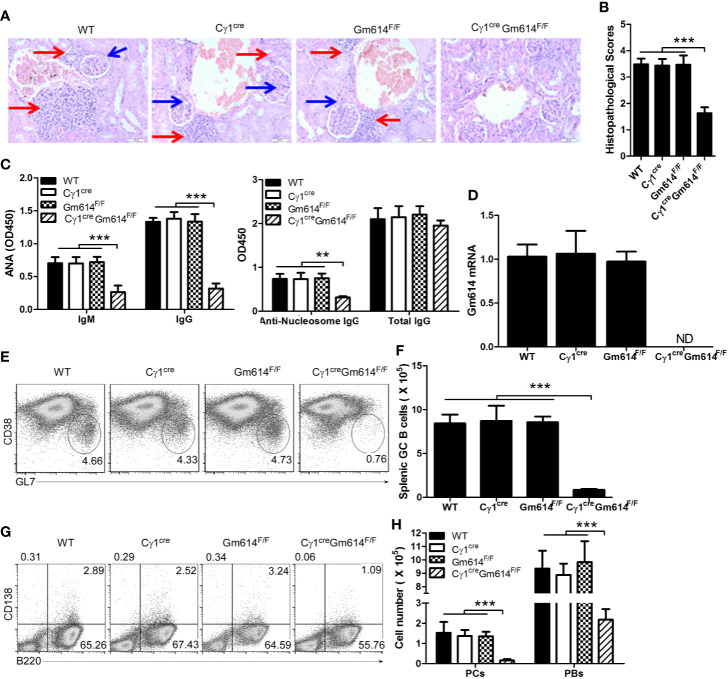
Gm614 knocked-out (KO) in GC B cells (Gm614 cKO) mitigated symptoms in pristane-induced lupus. Nine-week-old WT, C*γ*1^cre^ (C*γ*1-cre), Gm614^F/F^ (Gm614^fl/fl^), and C*γ*1^cre^Gm614^F/F^ (Gm614 cKO) mice were i.p. injected with 0.5 ml pristane and analyzed after 6 months. **(A)** Gm614 cKO reduced infiltrated inflammatory cells in the kidney of pristane-induced lupus. H&E-stained sections of the kidney. Scale bars, 50 µM. Blue arrows show glomeruli; red arrows show infiltrated inflammatory cells that appeared in the surrounding interstitial vasculature. **(B)** Gm614 cKO reduced histopathological scores of kidney. Histopathological scores were assessed based on loss of brush border, grading of tubular necrosis, tubular dilatation, and cast formation in 10 randomly chosen, non-overlapping fields. **(C)** Gm614 cKO reduced autoantibodies in pristane-induced lupus. Anti-nucleic antibodies (ANA) IgM and IgG, anti-nucleosome IgG, and total IgG were determined in the sera using ELISA assay. **(D)** Gm614 was depleted in Gm614 cKO mice. Splenic CD19^+^B220^+^CD38^lo^GL7^hi^ GC B cells were sorted by FACS and subject to qPCR. **(E, F)** Gm614 cKO reduced GC B cells in pristane-induced lupus. On gated of CD19^+^B220^+^ B cells, CD38^lo^GL7^hi^ GC B cells were analyzed by FACS. The percentages **(E)** and absolute numbers **(F)** of splenic GC B cells are shown. **(G, H)** Gm614 cKO reduced PBs and PCs in pristane-induced lupus. The percentages **(G)** and absolute numbers **(H)** of splenic CD138^+^B220^+^ PBs and CD138^-^B220^+^ PCs cells are shown. **(A–H)** Data represent three independent experiments, with six samples per group per experiment. **(B, D, F)** One-Way and **(C, H)** Two-Way ANOVA was followed by Bonferroni post-tests, Error bars, s.e.m., ***p < 0.001.

The hydrocarbon oil 2, 6, 10, 14-tetramethylpentadecane (TMPD; also known as pristane)-induced experimental lupus mice displayed some important immunologic and clinical features that are similar to those in human SLE (29–31). To explore the effect of Gm614 cKO on lupus progression, we injected pristane into WT and Gm614 cKO mice. H&E-staining of the mouse kidney sections at 6 months showed reduced inflammatory cell infiltration and overall normal structure of the glomerular region in Gm614 cKO mice **(****Figure 2A****)**. To evaluate the effect of Gm614 cKO on pristane-induced renal injury, histological scoring based on the typical microscopic features of chronic tubular damage, including extensive tubular necrosis and dilatation, as well as cast formation and loss of brush border was adopted. The results suggest that Gm614 cKO significantly protected the kidney from pristane-induced damage **(****Figure 2B****)**. Critically, Gm614 cKO reduced anti-nucleic antibodies (ANA) IgM and IgG, and anti-nucleosome IgG but not total IgG in pristane-induced lupus **(****Figure 2C****)**. Furthermore, we showed that Gm614 was depleted in the GC B cells of Gm614 cKO mice **(****Figure 2D****)**. This results in reduction in GC B cells **(****Figures 2E, F****)**, PBs, and PCs **(****Figures 2G, H****)** in Gm614 cKO mice. Taken together, these results suggest that Gm614 KO in GC B cells mitigates autoimmune symptoms by reducing GC B cells and subsequent PBs and PCs.

### Gm614 Transgene (Tg) in B Cells (BGm614 Tg) Exacerbated Autoimmune Symptoms

To further explore the effect of Gm614 overexpression on lupus, BM Chimeras were used to develop B-specific overexpression of Gm614 in B^Gm614 Tg^ mice **(****Supplementary Figure 2****)**. Overexpression of Gm614 was showed in B cells but not T cells **(****Supplementary Figure 2****)**; however, these mice did not demonstrate any open autoimmune symptoms.

**Figure 3:**
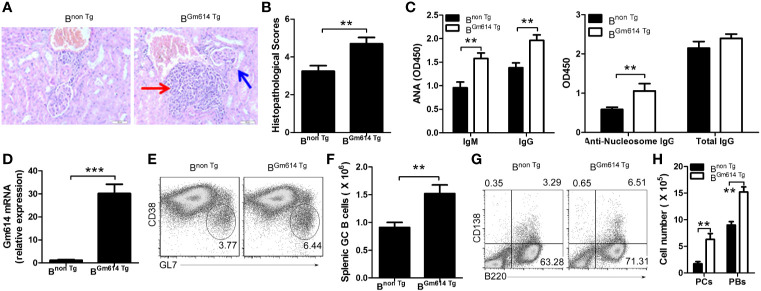
Gm614 transgene (Tg) in B cells (B^Gm614 Tg^) exacerbated symptoms in pristane-induced lupus. Nine-week-old B^non Tg^ and B^Gm614 Tg^ mice were i.p. injected with 0.5** **ml pristane and analyzed 6 months after injection. **(A)** Gm614 Tg promoted inflammatory cells to infiltrate into the kidney in pristane-induced lupus. H&E-stained sections of the kidney. Scale bars, 50 µM. Blue arrows show glomeruli; red arrows show infiltrated inflammatory cells that appeared in the surrounding interstitial vasculature. **(B)** Gm614 Tg up-regulated histopathological scores of kidney. Histopathological scores were assessed based on loss of brush border, grading of tubular necrosis, tubular dilatation, and cast formation in 10 randomly chosen, non-overlapping fields. **(C)** Gm614 Tg up-regulated autoantibodies in pristane-induced lupus. Anti-nucleic antibodies (ANA) IgM and IgG, anti-nucleosome IgG, and total IgG were determined in the sera using ELISA assay. **(D)** Gm614 was overexpression in B^Gm614 Tg^ mice. Splenic CD19^+^B220^+^CD38^lo^GL7^hi^ GC B cells were sorted by FACS and subject to qPCR. **(E, F)** Gm614 Tg up-regulated GC B cells in pristane-induced lupus. On gated CD19^+^B220^+^ B cells, CD38^lo^GL7^hi^ GC B cells were analyzed by FACS. The percentages **(E)** and absolute numbers **(F)** of splenic GC B cells are shown. **(G, H)** Gm614 Tg up-regulated PBs and PCs in pristane-induced lupus. The percentages **(G)** and absolute numbers **(H)** of splenic CD138^+^B220^+^ PBs and CD138^-^B220^+^ PCs cells are shown. **(A–H)** Data represent three independent experiments, with six samples per group per experiment. **(C, H)** Two-Way ANOVA was followed by Bonferroni post-tests and **(B, D, F)** Student’s t-test (two tailed), Error bars, s.e.m., **p < 0.01, ***p < 0.001.

To explore the effect of Gm614 overexpression on lupus progression, we injected pristane into B^non Tg^ and B^Gm614 Tg^ mice. H&E-stained sections of the kidney were analyzed 6 months after pristane injection. The results showed that Gm614 Tg promoted inflammatory cell infiltration into the kidney in pristane-induced lupus **(****Figure 3A****)**. To evaluate the effect of Gm614 Tg on pristane-induced renal injury, histological scoring based on the typical microscopic features of chronic tubular damage, including extensive tubular necrosis and dilatation, as well as cast formation and loss of brush border was adopted. The results suggest that Gm614 Tg significantly promotes pristane-induced damage in the kidney **(****Figure 3B****)**. In addition, anti-nucleic antibodies (ANA) IgM and IgG, and anti-nucleosome IgG but not total IgG were elevated in B^Gm614 Tg^ mice **(****Figure 3C****)**. This may result from Gm614 overexpression in GC B cells of these mice **(****Figure 3D****)**. Gm614 overexpression in GC B cells up-regulated GC B cells **(****Figures 3E, F****)**, PBs, and PCs **(****Figures 3G, H****)** in pristane-induced lupus. These results suggest that Gm614 overexpression in GC B cells exacerbated autoimmune symptoms by up-regulating GC B cells and subsequent PBs and PCs.

### Gm614 Protected GC B Cells From Death

**Figure 4:**
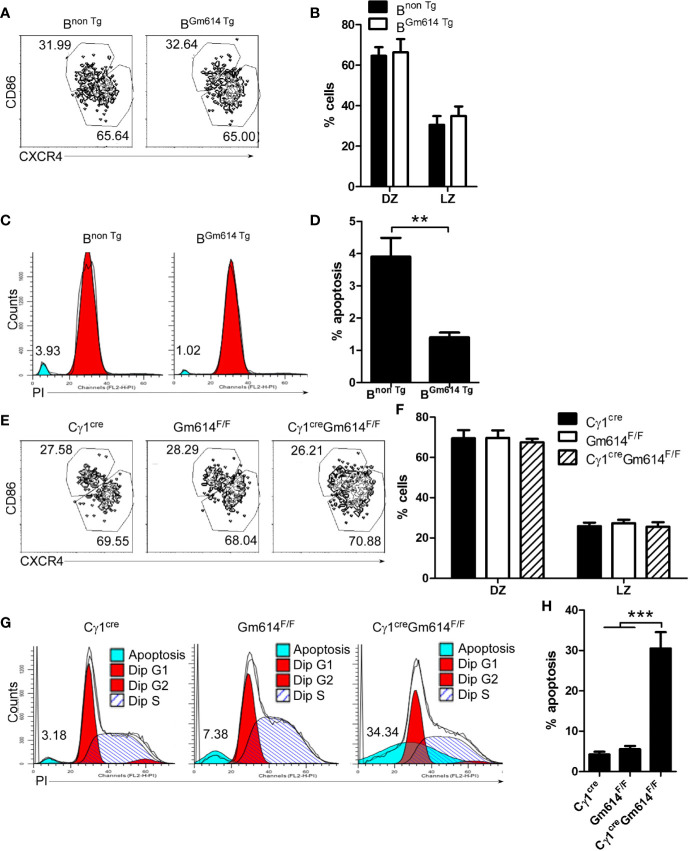
Gm614 suppressed GC B cell death. Nine-week-old B^non Tg^ and B^Gm614 Tg^ **(A–D)**, or C*γ*1^cre^, Gm614^F/F^, and C*γ*1^cre^Gm614^F/F^ **(E–H)** mice were i.p. injected with 0.5 ml pristane and analyzed 6 months after injection. **(A, B)** Gm614 Tg did not affect the percentages of B cells in dark zone (DZ) and light zone (LZ). On gated CD19^+^B220^+^CD38^lo^GL7^hi^ GC B cells, CD86^lo^CXCR4^hi^ DZ and CD86^hi^CXCR4^lo^ LZ B cells were analyzed by FACS. The percentages of splenic DZ and LZ B cells **(A)** and their statistical analysis **(B)** are shown. **(C, D)** Gm614 Tg suppressed GC B cell death. Splenic CD19^+^B220^+^CD38^lo^GL7^hi^ GC B cells were sorted by FACS and stained with PI (Propidium). The percentages of dead cells **(C)** and their statistical analysis **(D)** are shown. **(E, F)** Gm614 cKO did not affect the percentages of B cells in DZ and LZ. On gated CD19^+^B220^+^CD38^lo^GL7^hi^ GC B cells, CD86^lo^CXCR4^hi^ DZ and CD86^hi^CXCR4^lo^ LZ B cells were analyzed by FACS. The percentages of splenic DZ and LZ B cells **(E)** and their statistical analysis **(F)** are shown. **(G, H)** Gm614 cKO promoted GC B cell death. Splenic CD19^+^B220^+^CD38^lo^GL7^hi^ GC B cells were sorted by FACS and stained with PI. The percentages of dead cells **(G)** and their statistical analysis **(H)** are shown. **(A–H)** Data represent three independent experiments, with six samples per group per experiment. Two-Way **(B, F)** and One-Way **(H)** ANOVA was followed by Bonferroni post-tests and two tailed Student’s t-test **(D)**, Error bars, s.e.m., **p < 0.01, ***p < 0.001.

To explore the role of Gm614 in GC B cells, we first determined the effect of Gm614 on GC. We found that the percentages of CD86^lo^CXCR4^hi^ dark zone (DZ) and CD86^hi^CXCR4^lo^ light zone (LZ) B cells were unchanged between B^Gm614 Tg^ mice and B^non Tg^ mice **(****Figures 4A, B****)**. These results suggest that Gm614 Tg does not affect the ratio of B cells in DZ to LZ. Furthermore, we found that Gm614 Tg did not affect the GC B cell cycle but suppressed GC B cell death **(****Figures 4C, D****)**. On the other hand, we also used Gm614 cKO mice to detect the effect of Gm614 cKO on GC B cells. We found that Gm614 cKO did not affect the ratio of GC B cells in DZ to LZ **(****Figures 4E, F****)** and the cell cycle but promoted GC B cell death **(****Figures 4G, H****)**. These results demonstrate that Gm614 up-regulates GC B cells by protecting GC B cells from death.

### Gm614 Suppressed Caspase-1 Expression in GC B Cells

**Figure 5:**
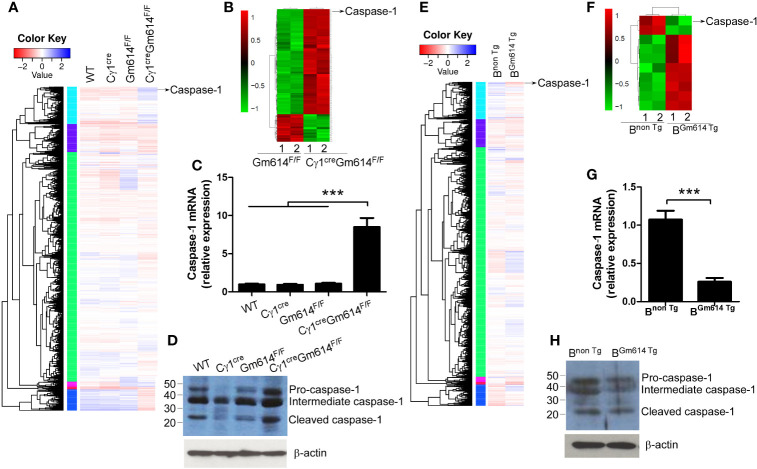
Gm614 suppressed caspase-1 expression in GC B cells. Nine-week-old WT, C*γ*1^cre^, Gm614^F/F^, and C*γ*1^cre^Gm614^F/F^ **(A–D)**, or B^non Tg^ and B^Gm614 Tg^ **(E–H)** mice were i.p. injected with 0.5 ml pristane and 6 months after injection, splenic CD19^+^B220^+^CD38^lo^GL7^hi^ GC B cells were sorted by FACS. **(A–D)** Gm614 cKO up-regulated caspase-1 expression in GC B cells. The transcripts and proteomics in GC B cells were determined by RNA-sequencing **(A)** and iTRAQ quantitative proteomics **(B)**, respectively, and caspase-1 is shown. Caspase-1 mRNA and protein expression in GC B cells were determined by qPCR **(C)** and western blot **(D)** assays, respectively. **(E–H)** Gm614 Tg suppressed caspase-1 expression in GC B cells. The transcripts and proteomics in GC B cells were determined by RNA-sequencing **(E)** and iTRAQ quantitative proteomics **(F)**, respectively, and caspase-1 is shown. Caspase-1 mRNA and protein expression in GC B cells were determined by qPCR **(G)** and western blot **(H)** assays, respectively. **(C, D, G, H)** Data represent three independent experiments, with six samples per group per experiment. **(C)** One-Way ANOVA was followed by Bonferroni post-tests and **(G)** Student’s t-test (two tailed), Error bars, s.e.m., ***p < 0.001.

To explore the mechanisms by which Gm614 protects GC B cells from death, we used RNA-sequencing and iTRAQ quantitative proteomics to determine the transcripts and proteomics in GC B cells, respectively. We found caspase-1 mRNA **(****Figure 5A****)** and protein **(****Figure 5B****)** were obviously increased in Gm614^-/-^GC B cells. These results were further proven by qPCR **(****Figure 5C****)** and western blot **(****Figure 5D****)** assays. Our data suggest that Gm614 cKO up-regulated caspase-1 expression in GC B cells. On the other hand, we also used RNA-sequencing **(****Figure 5E****)** and iTRAQ quantitative proteomics to determine the effect of Gm614 Tg on the transcripts and proteomics in GC B cells. Gm614 Tg obviously reduced caspase-1 mRNA **(****Figure 5E****)** and protein **(****Figure 5F****)** in GC B cells. This was subsequently proven by qPCR **(****Figure 5G****)** and western blot **(****Figure 5H****)** assays. Our results suggest that Gm614 suppressed caspase-1 expression in GC B cells.

### Gm614 Suppressed Caspase-1 Transcription by Binding With the Caspase-1 Promoter

**Figure 6:**
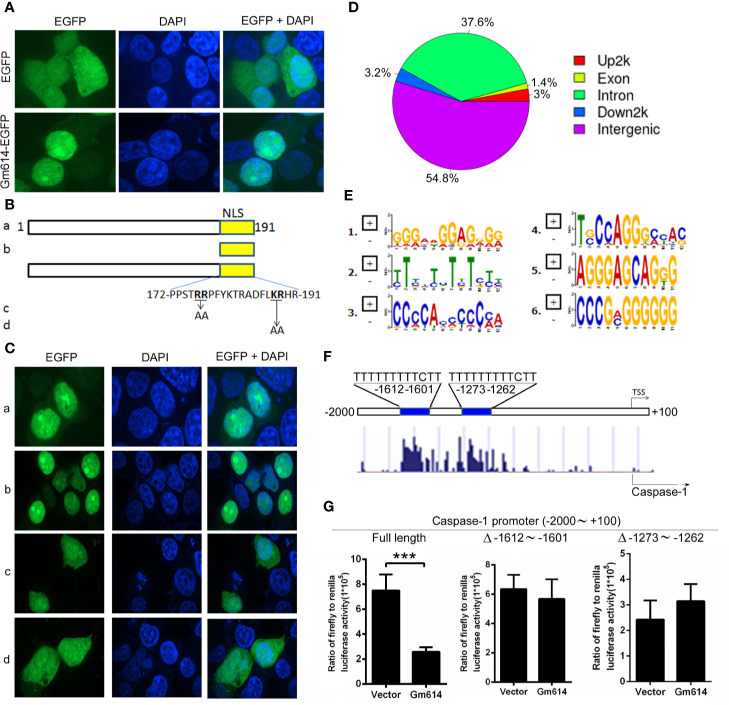
Gm614 suppressed caspase-1 transcription. **(A)** Gm614 was expressed in the nucleus. CD19^+^B220^+^CD38^lo^GL7^hi^ GC B cells were infected with lentiviruses with EGFP- or Gm614-EGFP-expressing LV122 and cultured for 2 days. Cells were imaged and analyzed on a GE IN Cell Analyzer 2000. Representative images show the nuclear location of Gm614. **(B, C)** Nuclear localization sequence (NLS) was located in C-terminal (172∼191) of Gm614. LV122 lentiviruses expressing **(A)** full length (1–191)-EGFP, **(b)** NLS (172–191)-EGFP, **(c)** full length with AA (176–177) mutation-EGFP, or **(d)** full length with AA (188–189) mutation-EGFP **(B)** were infected into CD19^+^B220^+^CD38^lo^GL7^hi^ GC B cells and on day 2, cells were imaged on a GE IN Cell Analyzer 2000 **(C)**. **(D–F)** Gm614 bound with the caspase-1 promoter. CD19^+^B220^+^CD38^lo^GL7^hi^ GC B cells were infected with lentiviruses containing EGFP- or Gm614-EGFP-expressing LV122, and cultured for 3 days. Genome-wide mapping of Gm614 binding in GC B cells by ChIP-seq. **(D)** Distribution of Gm614-binding peaks. **(E)** De novo motif prediction by DNA sequences enriched in Gm614 binding regions. **(F)** Genomic snapshots depicting the ChIP-seq results for Gm614 (lower panel) and the predicted motif (upper panel) at the promoter regions of the caspase-1 genomic loci. **(G)** Gm614 suppressed the activation of caspase-1 promoter. Gm614-expressing LV201 (Gm614) or empty vector LV 201 (Vector) and luciferase reporter vector pEZX-PG04.1/caspase-1 promoter (-2000 ∼ +100 bp of mouse caspase-1 gene) (Full length), caspase-1 promoter with the deletion of -1612 ∼ -1601 (Δ -1612 ∼ -1601) or -1273 ∼ -1262 (Δ -1273 ∼ -1262) were co-transduced into 293T cells. Dual luciferase reporter gene expression was analyzed, and the results are shown as the ratio of firefly to Renilla luciferase activity. **(A, C, G)** Data represent three independent experiments, with six samples per group per experiment. **(G)** Student’s t-test (two tailed), Error bars, s.e.m., ***p < 0.001.

To explore the mechanisms by which Gm614 suppressed caspase-1 expression in GC B cells, we first determined the subcellular location of Gm614. We found that Gm614 was expressed in the nucleus **(****Figure 6A****)**. Further, we found that nuclear localization sequence (NLS) was located in the C-terminus (172∼191) of Gm614 **(****Figures 6B, C****)**. We proposed that Gm614 could bind with DNA. Genome-wide mapping of Gm614 binding in GC B cells using ChIP-seq showed that Gm614 could bind Up2k, which is often contained within the promoter **(****Figure 6D****)**. De novo motif prediction by DNA sequences enriched in Gm614 binding regions demonstrated 6 Gm614 binding motifs **(****Figure 6E****)**. Genomic snapshots depicting the ChIP-seq results showed two Gm614 binding sites (-1612 ∼ -1601, -1273 ∼ -1262) at the caspase-1 genomic loci promoter regions (**Figure 6F**, lower panel). Critically, these two sites were the same sequences (TTTTTTTTTCTT) as the second motif suggested by *de novo* motif prediction (**Figure 6F**, upper panel). These results indicate that Gm614 could bind with the promoter of caspase-1. Dual luciferase reporter gene expression was analyzed to examine the effect of Gm614 on the caspase-1 promoter and we found that Gm614 could effectively suppress its activation (**Figure 6G**). However, Gm614 did not suppress the activation of caspase-1 promoters with deletions at the -1612 ∼ -1601 or -1273 ∼ -1262 sites that binds Gm614 **(****Figure 6G****)**. These results suggest that Gm614 suppressed caspase-1 transcription by binding with the caspase-1 promoter.

### Gm614 Promoted KLH-Induced GC B-Cell Responses

**Figure 7:**
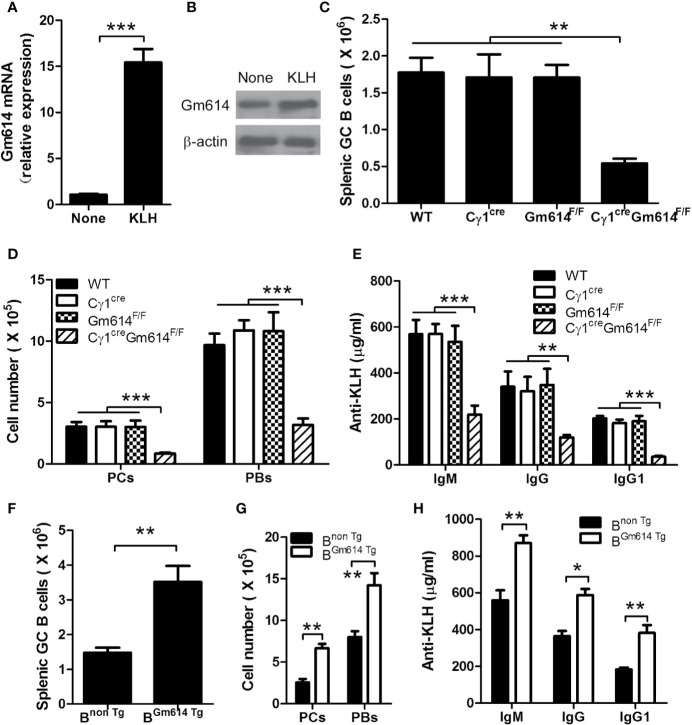
Gm614 up-regulated GC B-cell responses induced by foreign antigen KLH. Nine-week-old WT, C*γ*1^cre^ (C*γ*1-cre), Gm614^F/F^ (Gm614^fl/fl^), and C*γ*1^cre^Gm614^F/F^ (Gm614 cKO), or B^non Tg^ and B^Gm614 Tg^ mice were i.p. injected with 100 μg of the protein antigen KLH in a volume of 0.2 mL of saline and analyzed after 28 days. **(A, B)** Gm614 expression was up-regulated in GC B cells by foreign antigen KLH. GC B cells from splenocytes of six nonimmunized WT mice and KLH-immunized WT mice were sorted by FACS, and subjected to qPCR **(A)** and western blot **(B)** analysis. **(C–E)** Gm614 cKO suppressed GC B-cell responses induced by KLH. Splenocytes and sera were collected from KLH-immunized WT, C*γ*1^cre^, Gm614^F/F^, and C*γ*1^cre^Gm614^F/F^ mice. Splenic CD19^+^B220^+^CD38^lo^GL7^hi^ GC B cells, CD138^+^B220^+^ PBs, and CD138^-^B220^+^ PCs cells were analyzed and the absolute number of GC B cells **(C)**, and PBs and PCs **(D)** was shown. Anti-KLH IgM, IgG, and IgG1 were determined in the sera using ELISA assay **(E). (F–H)** Gm614 Tg promoted GC B-cell responses induced by KLH. Splenocytes and sera were collected from KLH-immunized B^non Tg^ and B^Gm614 Tg^ mice. Splenic CD19^+^B220^+^CD38^lo^GL7^hi^ GC B cells, CD138^+^B220^+^ PBs, and CD138^-^B220^+^ PCs cells were analyzed and the absolute number of GC B cells **(F)**, and PBs and PCs **(G)** was shown. Anti-KLH IgM, IgG, and IgG1 were determined in the sera using ELISA assay **(H)**. **(A–H)** Data represent three independent experiments, with six samples per group per experiment. Two-Way **(D, E, G, H)** and One-Way **(C)** ANOVA was followed by Bonferroni post-tests and two tailed Student’s t-test **(A, F)**, Error bars, s.e.m., *p < 0.05, **p < 0.01, ***p < 0.001.

To study whether a foreign antigen promoted GC B cells to express Gm614, we determined the expression of Gm614 in spontaneous GCs of WT mice and KLH-immunized WT mice. We found that Gm614 expression was up-regulated in GC B cells by foreign antigen KLH **(****Figures 7A, B****)**. To further explore whether Gm614 plays an important role in an optimal GC responses induced by an foreign antigen, we examined splenic CD19^+^B220^+^CD38^lo^GL7^hi^ GC B cells, CD138^+^B220^+^ PBs, and CD138^-^B220^+^ PCs cells and anti-KLH IgM, IgG, and IgG1 in the sera from KLH-immunized WT, C*γ*1^cre^, Gm614^F/F^, and C*γ*1^cre^Gm614^F/F^ mice. We found that Gm614 cKO reduced the absolute number of GC B cells **(****Figure 7C****)**, PBs and PCs **(****Figure 7D****)**, anti-KLH IgM, IgG, and IgG1 antibodies **(****Figure 7E****)** induced by KLH. These results suggest that Gm614 cKO suppressed KLH-induced GC B-cell responses. In addition, we also determined splenic CD19^+^B220^+^CD38^lo^GL7^hi^ GC B cells, CD138^+^B220^+^ PBs, and CD138^-^B220^+^ PCs cells and anti-KLH IgM, IgG, and IgG1 in the sera from KLH-immunized B^non Tg^ and B^Gm614 Tg^ mice. Our data demonstrated that Gm614 Tg up-regulated the absolute number of GC B cells **(****Figure 7F****)**, PBs and PCs **(****Figure 7G****)**, anti-KLH IgM, IgG, and IgG1 antibodies **(****Figure 7H****)** induced by KLH. These results suggest that Gm614 Tg promoted GC B-cell responses induced by KLH.

## Discussion

Germinal center (GC) B-cell proliferation, differentiation, survival, and death are strictly regulated by many molecules including Bcl6 and Aid. The transcriptional repressor Bcl6 directly controls many GC B cell functions including activation, survival, DNA damage response, cell cycle arrest, cytokines, toll-like receptors, TGF-β, WNT signaling, and so on (32). In GC, immunoglobulin (Ig) genes in Ag-activated B cells undergo somatic hypermutation (SHM) and class switching recombination (CSR) initiated by Aid (33, 34). Apart from Bcl6 and Aicda (Aid), Gm614 is highly expressed in GC B cells from lupus-prone mice **(****Figure 1****)**. In addition, Gm614 protects GC B cells from death **(****Figure 4****)**, and the expression of Gm614 in GC B cells of lupus-prone mice plays an important role in the balance between GC B-cell survival and death.

Cell-intrinsic induction of Bcl-6 has been shown to promote autoimmune germinal centers (35). In addition, increased Aid expression is also associated with some autoimmune diseases, such as SLE (36). We demonstrated here that lupus could up-regulate Gm614 expression in GC B cells **(****Figure 1****)**. This increased Gm614 expression could effectively up-regulate GC B cells, resulting in increased PBs and PCs, and finally exacerbated autoimmune symptoms **(****Figure 3****)**, whereas Gm614 cKO effectively reduced GC B cells, resulted in decreased PBs and PCs and mitigation of autoimmune symptoms **(****Figure 2****)**. These results suggest that similar to Bcl-6 and Aid, Gm614 also has a critical role in autoimmune diseases.

Dark zones in mature GC contain mainly proliferating cells, and some apoptotic B cells and non-selected B cells also undergo apoptosis in light zones of GC (37). The up-regulation of B7-H1 on activated B cells suggests the possibility that PD-1/B7-H1 interactions may regulate survival of self-reactive B cells during GC reactions (38–40). We showed here that up-regulation of Gm614 protected GC B cells from apoptosis or death **(****Figure 4****)**. Because apoptosis plays critical roles in the initiation, progression, and remission of autoimmune diseases, it is the major target of therapeutic interventions for autoimmune diseases (41). For example, induction of GC B-cell apoptosis by targeting Gm614 may be a potential way to control autoimmune diseases.

Activated caspase-1 induces pyroptosis, inflammatory and necrosis-like programmed cell death (42). However, interleukin (IL)-1β and IL-18 secretion is rare in GC B cells. Thus, the main activity of caspase-1 is to induce B cell death (42). Critically, it has been shown that caspase-1 regulates splenic B-cell apoptosis independent of IL-1β and IL-18 (43). Our data here showed that GC B cell apoptosis or death **(****Figure 4****)** is positively associated with caspase-1 expression **(****Figure 5****)**. Thus, Gm614-mediated suppression of caspase-1 expression **(****Figure 5****)** is at least partly involved in the imbalance between B-cell proliferation and death in autoimmune diseases.

Mechanistically, we showed that Gm614 is located in the nucleus of GC B cells **(****Figure 6A****)**. Further, we found a nuclear localization sequence (NLS) in the C-terminal (172∼191) of Gm614 **(****Figures 6B, C****)**. In addition, we found that amino acids (AAs) in 176-RR-177 and 188-KR-189 are critical for NLS. These AAs have been reported as critical residues of NLS (44). Online (http://mleg.cse.sc.edu/seqNLS/) prediction showed that 176-RRPFYKTRADFLKRHR-192 had a very high score (0.949), which is in line with our experimental results. Furthermore, we showed that Gm614 suppressed caspase-1 transcription by binding with two sites (-1612 ∼ -1601, -1273 ∼ -1262) of the caspase-1 promoter **(****Figures 6F, G****)**. Apart from Up2k, exon, intron down2k, and intergenes are also bound by Gm614 **(****Figure 6D****)**. This suggests that Gm614 may have many other functions.

Apart from the role of Gm614 in the autoimmune environment, we also explored the role of Gm614 in GC B cells in general. We found that foreign antigens (e.g., KLH) could up-regulate Gm614 expression in GC B cells **(****Figures 7A, B****)**. Furthermore, Gm614 cKO suppressed KLH-induced GC B-cell responses **(****Figure 7C****)**, which resulted in reduced PBs and PCs **(****Figure 7D****)** and anti-KLH IgM, IgG, and IgG1 antibodies **(****Figure 7E****)**. In addition, we showed that Gm614 Tg promoted KLH-induced GC B-cell responses **(****Figure 7F****)**, which resulted in increased PBs and PCs **(****Figure 7G****)** and anti-KLH IgM, IgG, and IgG1 antibodies **(****Figure 7H****)**. These results define the role of Gm614 in GC B cells in general. Thus, Gm614 is required for GC B cell survival irrespective of an autoimmune or non-autoimmune environment. This will have implication in vaccine development. In conclusion, we showed that a novel uncharacterized Gm614, highly expressed in GC B cells of lupus-prone mice, protected GC B cells from death and exacerbated autoimmune symptoms by binding with caspase-1 promoter to suppress its activation. Thus, our results provide a novel mechanism underlying the imbalance between B-cell proliferation and death in autoimmune diseases and provides some hints for treatment of autoimmune diseases by targeting Gm614.

## Data Availability Statement

The datasets presented in this study can be found in online repositories. The names of the repository/repositories and accession number(s) can be found in the article/**Supplementary Material**.

## Ethics Statement

The animal study was reviewed and approved by the Animal Ethics Committee of Beijing Institute of Brain Disorders.

## Author Contributions

YH, RX, BZ, and SZ performed the experiments, and XW contributed essential reagents and materials for the experiments. RW conceived and designed the studies. YH, RX, and RW contributed to data analysis and manuscript preparation. All authors contributed to the article and approved the submitted version.

## Funding

This study was supported by grants from National Natural Science Foundation of China (82071758, 31770956, 81704151), Natural Science Foundation of Beijing Municipality (7182121), Beijing Municipal Commission of Education (KM201710025014), and China Postdoctoral Science Foundation Funded Project (2019M664000).

## Conflict of Interest

Author XW was employed by the company Staidson (Beijing) Biopharmaceuticals.

The remaining authors declare that the research was conducted in the absence of any commercial or financial relationships that could be construed as a potential conflict of interest.

## References

[1] Christiane SH B Cells in Autoimmune Diseases. Sci (Cairo) (2012) 2012:215308. 10.6064/2012/215308

[2] Hofmann K Clauder AK Manz RA Targeting B Cells and Plasma Cells in Autoimmune Diseases. Front Immunol (2018) 9:835. 10.3389/fimmu.2018.00835 29740441 PMC5924791

[3] Rieux-Laucat F Le Deist F Fischer A Autoimmune lymphoproliferative syndromes: genetic defects of apoptosis pathways. Cell Death Differ (2003) 10(1):124–33. 10.1038/sj.cdd.4401190 12655301

[4] Chu JL Drappa J Parnassa A Elkon KB The defect in Fas mRNA expression in MRL/lpr mice is associated with insertion of the retrotransposon, ETn. J Exp Med (1993) 178(2):723–30. 10.1084/jem.178.2.723 PMC2191101 7688033

[5] Macanovic M Sinicropi D Shak S Baughman S Thiru S Lachmann PJ The treatment of systemic lupus erythematosus (SLE) in NZB/W F1 hybrid mice; studies with recombinant murine DNase and with dexamethasone. Clin Exp Immunol (1996) 106(2):243–52. 10.1046/j.1365-2249.1996.d01-839.x PMC2200591 8918569

[6] Luzina IG Atamas SP Storrer CE daSilva LC Kelsoe G Papadimitriou JC Spontaneous formation of germinal centers in autoimmune mice. J Leukoc Biol (2001) 70(4):578–84. 10.1189/jlb.70.4.578 11590194

[7] Dorner T Giesecke C Lipsky PE Mechanisms of B cell autoimmunity in SLE. Arthritis Res Ther (2011) 13(5):243. 10.1186/ar3433 22078750 PMC3308063

[8] Cassese G Lindenau S de Boer B Arce S Hauser A Riemekasten G Inflamed kidneys of NZB / W mice are a major site for the homeostasis of plasma cells. Eur J Immunol (2001) 31(9):2726–32. 10.1002/1521-4141(200109)31:9<2726::AID-IMMU2726>3.0.CO;2-H 11536171

[9] Victora GD Nussenzweig MC Germinal centers. Annu Rev Immunol (2012) 30:429–57. 10.1146/annurev-immunol-020711-075032 22224772

[10] Wang X Zhang Y Wang Z Liu X Zhu G Han G Anti-IL-39 (IL23p19/Ebi3) polyclonal antibodies ameliorate autoimmune symptoms in lupus-like mice. Mol Med Rep (2018) 17:1660–6. 10.3892/mmr.2017.8048 PMC5780108 29138852

[11] Ma N Xing C Xiao H He Y Han G Chen G BAFF Suppresses IL-15 Expression in B Cells. J Immunol (2014) 192:4192–201. 10.4049/jimmunol.1302132 24670802

[12] Dominguez-Sola D Kung J Holmes AB Wells VA Mo T Basso K The FOXO1 Transcription Factor Instructs the Germinal Center Dark Zone Program. Immunity (2015) 43(6):1064–74. 10.1016/j.immuni.2015.10.015 26620759

[13] Sander S Chu VT Yasuda T Franklin A Graf R Calado DP PI3 Kinase and FOXO1 Transcription Factor Activity Differentially Control B Cells in the Germinal Center Light and Dark Zones. Immunity (2015) 43(6):1075–86. 10.1016/j.immuni.2015.10.021 26620760

[14] Fillatreau S Sweenie CH McGeachy MJ Gray D Anderton SM B cells regulate autoimmunity by provision of IL-10. Nat Immunol (2002) 3(10):944–50. 10.1038/ni833 12244307

[15] Fillatreau S Gray D T cell accumulation in B cell follicles is regulated by dendritic cells and is independent of B cell activation. J Exp Med (2003) 197(2):195–206. 10.1084/jem.20021750 12538659 PMC2193813

[16] Recaldin T Fear DJ Transcription factors regulating B cell fate in the germinal centre. Clin Exp Immunol (2016) 183(1):65–75. 10.1111/cei.12702 26352785 PMC4687514

[17] Tellier J Nutt SL Standing out from the crowd: How to identify plasma cells. Eur J Immunol (2017) 47(8):1276–9. 10.1002/eji.201747168 28787106

[18] Igarashi K Ochiai K Muto A Architecture and dynamics of the transcription factor network that regulates B-to-plasma cell differentiation. J Biochem (2007) 141(6):783–9. 10.1093/jb/mvm106 17569706

[19] Fang Y Xu R Zhai B Hou C Ma N Wang L Gm40600 suppressed SP 2/0 isograft tumor by reducing Blimp1 and Xbp1 proteins. BMC Cancer (2019) 19(1):700. 10.1186/s12885-019-5848-1 31311517 PMC6636126

[20] Xu R Fang Y Hou C Zhai B Jiang Z Ma N BC094916 suppressed SP 2/0 xenograft tumor by down-regulating Creb1 and Bcl2 transcription. Cancer Cell Int (2018) 18:138. 10.1186/s12935-018-0635-7 30220882 PMC6137751

[21] Zhai B Hou C Xu R Fang Y Xiao H Chen G Loc108167440 suppressed myeloma cell growth by p53-mediated apoptosis. Leuk Lymphoma (2019) 60(10):2541–8. 10.1080/10428194.2019.1590572 30947584

[22] Zhai B Hou C Xu R Fang Y Ma N Xing C Gm6377 suppressed SP 2/0 xenograft tumor by down-regulating Myc transcription. Clin Translat Oncol (2020) 22(9):1463–71. 10.1007/s12094-019-02280-y 31950438

[23] Xu R Wang R Han G Wang J Chen G Wang L Complement C5a regulates interleukine-17 by affecting the crosstalk between DCs and gammadelta T cells in CLP-induced sepsis. Eur J Immunol (2010) 40:1079–88. 10.1002/eji.200940015 20140904

[24] Xing C Ma N Xiao H Wang X Zheng M Han G Critical role for thymic CD19^+^CD5^+^CD1d^hi^IL-10^+^ regulatory B cells in immune homeostasis. J Leukoc Biol (2015) 97(3):547–56. 10.1189/jlb.3A0414-213RR PMC5477891 25516754

[25] Ma N Liu X Xing C Wang X Wei Y Han G Ligation of metabotropic glutamate receptor 3 (Grm3) ameliorates lupus-like disease by reducing B cells. Clin Immunol (2015) 160(2):142–54. 10.1016/j.clim.2015.05.016 26071318

[26] Melnikov VY Faubel S Siegmund B Lucia MS Ljubanovic D Edelstein CL Neutrophil-independent mechanisms of caspase-1- and IL-18-mediated ischemic acute tubular necrosis in mice. J Clin Invest (2002) 110:1083–91. 10.1172/JCI0215623 PMC150794 12393844

[27] Zhang Y Wang Z Xiao H Liu X Zhu G Yu D Foxd3 suppresses IL-10 expression in B cells. Immunology (2017) 150(4):478–88. 10.1111/imm.12701 PMC5343362 27995618

[28] Ma N Xiao H Marrero B Xing C Wang X Zheng M Combination of TACI-IgG and Anti-IL-15 Treats Murine Lupus by reducing mature and memory B cells. Cell Immunol (2014) 289:140–4. 10.1016/j.cellimm.2014.03.017 24791699

[29] Freitas EC de Oliveira MS Monticielo OA Pristane-induced lupus: considerations on this experimental model. Clin Rheumatol (2017) 36(11):2403–14. 10.1007/s10067-017-3811-6 28879482

[30] Leiss H Niederreiter B Bandur T Schwarzecker B Bluml S Steiner G Pristane-induced lupus as a model of human lupus arthritis: evolvement of autoantibodies, internal organ and joint inflammation. Lupus (2013) 22(8):778–92. 10.1177/0961203313492869 23817510

[31] Wang X Wei Y Xiao H Liu X Zhang Y Han G Pre-existing CD19-independent GL7- Breg cells are expanded during inflammation and in mice with lupus-like disease. Mol Immunol (2016) 71:54–63. 10.1016/j.molimm.2016.01.011 26852110 PMC11315234

[32] Basso K Dalla-Favera R Roles of BCL6 in normal and transformed germinal center B cells. Immunol Rev (2012) 247(1):172–83. 10.1111/j.1600-065X.2012.01112.x 22500840

[33] Chandra V Bortnick A Murre C AID targeting: old mysteries and new challenges. Trends Immunol (2015) 36(9):527–35. 10.1016/j.it.2015.07.003 PMC4567449 26254147

[34] Okazaki IM Kotani A Honjo T Role of AID in tumorigenesis. Adv Immunol (2007) 94:245–73. 10.1016/S0065-2776(06)94008-5 17560277

[35] Jackson SW Jacobs HM Arkatkar T Dam EM Scharping NE Kolhatkar NS B cell IFN-γ receptor signaling promotes autoimmune germinal centers via cell-intrinsic induction of BCL-6. J Exp Med (2016) 213(5):733–50. 10.1084/jem.20151724 PMC4854732 27069113

[36] Incorvaia E Sicouri L Petersen-Mahrt SK Schmitz KM Hormones and AID: balancing immunity and autoimmunity. Autoimmunity (2013) 46(2):128–37. 10.3109/08916934.2012.748752 23181348

[37] Zhang Y Garcia-Ibanez L Toellner KM Regulation of germinal center B-cell differentiation. Immunol Rev (2016) 270(1):8–19. 10.1111/imr.12396 26864101 PMC4755139

[38] Chtanova T Tangye SG Newton R Frank N Hodge MR Rolph MS T follicular helper cells express a distinctive transcriptional profile, reflecting their role as non-Th1/Th2 effector cells that provide help for B cells. J Immunol (2004) 173:68–78. 10.4049/jimmunol.173.1.68 15210760

[39] Klein U Dalla-Favera R Germinal centres: role in B-cell physiology and malignancy. Nat Rev Immunol (2008) 8:22–33. 10.1038/nri2217 18097447

[40] Keir ME Butte MJ Freeman GJ Sharpe AH PD-1 and its ligands in tolerance and immunity. Annu Rev Immunol (2008) 26:677–704. 10.1146/annurev.immunol.26.021607.090331 18173375 PMC10637733

[41] Nagata S Apoptosis and autoimmune diseases. Ann N Y Acad Sci (2010) 1209:10–6. 10.1111/j.1749-6632.2010.05749.x 20958310

[42] Miao EA Rajan JV Aderem A Caspase-1 induced pyroptotic cell death. Immunol Rev (2011) 243(1):206–14. 10.1111/j.1600-065X.2011.01044.x PMC3609431 21884178

[43] Sarkar A Hall MW Exline M Hart J Knatz N Gatson NT Caspase-1 regulates Escherichia coli sepsis and splenic B cell apoptosis independently of interleukin-1beta and interleukin-18. Am J Respir Crit Care Med (2006) 174(9):1003–10. 10.1164/rccm.200604-546OC PMC2648100 16908867

[44] Kalderon D Roberts BL Richardson WD Smith AE A short amino acid sequence able to specify nuclear location. Cell (1984) 39(3 Pt 2):499–509. 10.1016/0092-8674(84)90457-4 6096007

