# Supplementary material for: Gm614 Protects Germinal Center B Cells From Death by Suppressing Caspase-1 Transcription in Lupus-Prone Mice
Source: Front Immunol. 2020 Oct 21;11:585726. doi: 10.3389/fimmu.2020.585726 (PMC7609865; doi:10.3389/fimmu.2020.585726)
Supplement: Supplementary file 1 [file DataSheet_1.pdf]

Supplementary Figure 1

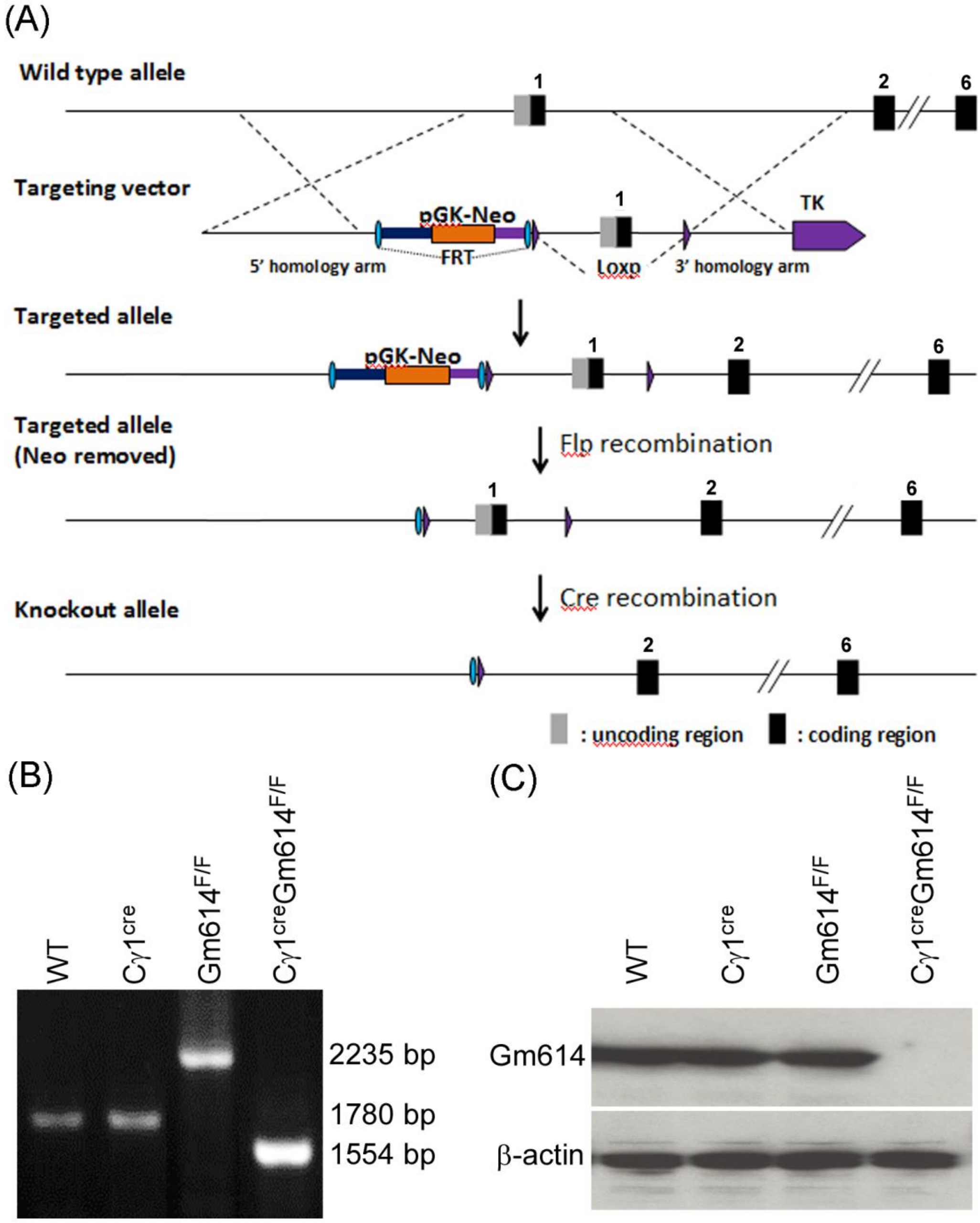

Supplementary figure 1. Cγ1^{cre}Gm614$^{fl/fl}$ (Cγ1^{cre}Gm614$^{F/F}$) mice were developed. (A) A construction map of Gm614$^{F/F}$ and Cγ1^{cre}Gm614$^{F/F}$ mice. This project used the principle of homologous recombination and adopted embryonic stem (ES) cell targeting to modify the Gm614 locus by flox modification. The brief process is as follows: The BAC clone containing the gene of interest was purchased from the Sanger Institute (UK). The ES cell targeting vector was constructed by the ET-clone

method. The vector contains a 3.4Kb 5’ homology arm, a 541bp flox region, and a PGK-neo-polyA, 3.6kb 3’ homology arm, plus MC1-TK-polyA negative selection marker. After the vector was linearized, JM8A3 ES cells were transfected electrically. A total of 96 resistant clones were obtained after screening with the G418 and Ganc drugs. The positive clones with correct homologous recombination were identified by long fragment PCR. Positive ES cell clones were expanded and injected into blastocysts of C57BL/6J mice to obtain chimeric mice. A high proportion of chimeric mice were mated with C57BL/6J mice to obtain seven positive F1 mice. Gm614 gene flox heterozygous mice showed no significant abnormalities. After mating the flox mouse with a heterologous $C\gamma1^{cre}$ mouse, the progeny of the flox homozygous, Cre-positive mouse was knocked out, resulting in a functional loss of the gene of interest in GC B cells. **(B, C) Gm614 was knocked out in GC B cells from $C\gamma1^{cre}Gm614^{F/F}$ mice.** GC B cells ($CD19^{+}B220^{+}CD38^{low}GL7^{hi}$) from the spleens of 7- to 9-week-old wild type (WT), heterologous $C\gamma1^{cre}$, $Gm614^{F/F}$, and $C\gamma1^{cre}Gm614^{F/F}$ mice were sorted by FACS. GC B cells were subjected to PCR (**B**) and western blot (**C**) analysis. PCR products: with cre activity: 1554 bp; with no cre activity: 2235 bp; wild type：1780 bp. (**B, C**) Data represent three independent experiments.

Supplementary figure 2

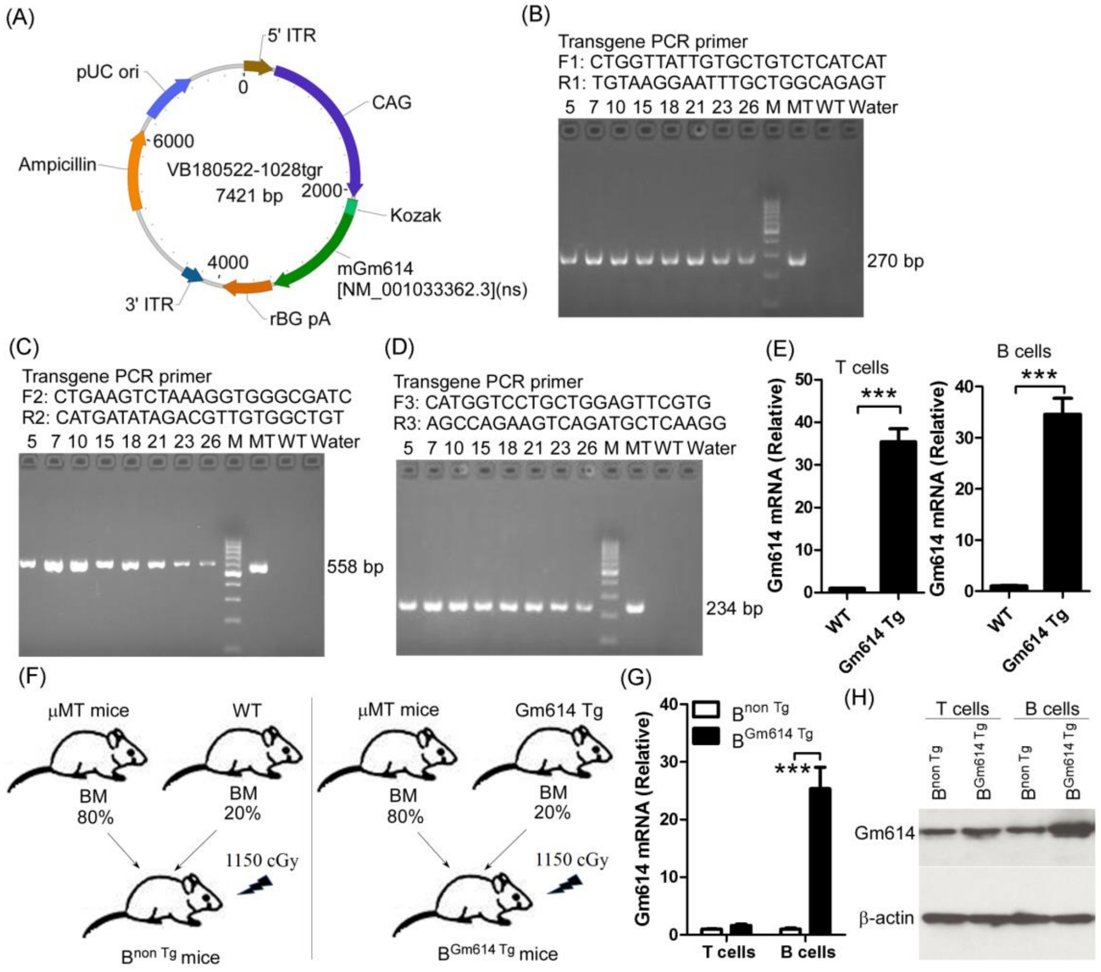

Supplementary figure 2. The construction and identification of Gm614 Tg mice and B$^{Gm614\ Tg}$ mice. (A) This vector is for making transgenic mice. Vector name: VB180522-1028tgr; Size: 7421 bp; Vector Type: PiggyBac transposon gene expression vector; Inserted Promoter: CAG; Inserted ORF: mGm614 [NM_001033362.3] (ns). **(B-D) Gm614 Tg mice were identified by PCR assay.** We used TaKaRa MiniBEST Universal Genomic DNA Extraction kit (Ver.5.0_Code No. 9765) to gain high purity of genomic DNA. 3 sets of primers for identification of transgenic Gm614 were shown in the upper of gels. 8 mice (No. 5, 7, 10, 15, 18, 21, 23, and 26) were identified positive. M: Marker. MT: Positive control, 400 ng of mouse genomic DNA spiked with an amount of transgene injection DNA that is

equivalent to 5 copies of transgene per diploid mouse genome. WT: Wild type mice. Water: negative control for PCR. **(E) Gm614 was overexpressed in CD3^{+}CD4^{+}T cells and B220^{+}CD19^{+}B cells from Gm614 Tg mice.** Splenocytes from 9-week-old WT and Gm614 Tg mice were stained with fluorescence-conjugated anti-mouse CD3 and CD4 antibodies or B220 and CD19 antibodies. CD3^{+}CD4^{+}T cells and B220^{+}CD19^{+}B cells were sorted by FACS and subject to q-PCR. **(F) A construction map of B$^{non\ Tg}$ and B$^{Gm614\ Tg}$ mice.** μMT mice were irradiated (1150 cGy) and reconstituted with BM from μMT mice (80%) and Gm614 Tg mice (20%). Thus, 80% of the hematopoietic cells (except B cells) in the chimeric mice will be wild-type in gene expression whereas the B cells can only be derived from Gm614 Tg precursors. The reconstituted mice were named as B$^{Gm614\ Tg}$ mice. B$^{non\ Tg}$ mice were used as the control. **(G, H) Gm614 was overexpressed in B220^{+}CD19^{+}B cells but not CD3^{+}CD4^{+}T cells from B$^{Gm614\ Tg}$ mice.** Splenocytes from 9-week-old B$^{Gm614\ Tg}$ and B$^{non\ Tg}$ mice were stained with fluorescence-conjugated anti-mouse CD3 and CD4 antibodies or B220 and CD19 antibodies. CD3^{+}CD4^{+}T cells and B220^{+}CD19^{+}B cells were sorted by FACS and subject to q-PCR (G) and western blot (H). (E, G) Data represent three independent experiments, with six individual mice per group per experiment and were analyzed by two tailed Student's t-test (E) and two-way ANOVA were followed by Bonferroni post-tests (G). Error bars represent s.e.m. ***$P < 0.001$.
